# Effects of a three-armed randomised controlled trial using self-monitoring of daily steps with and without counselling in prediabetes and type 2 diabetes—the Sophia Step Study

**DOI:** 10.1186/s12966-021-01193-w

**Published:** 2021-09-08

**Authors:** Jenny Rossen, Kristina Larsson, Maria Hagströmer, Agneta Yngve, Kerstin Brismar, Barbara Ainsworth, Linda Åberg, Unn-Britt Johansson

**Affiliations:** 1 Department of Health Promoting Science, Sophiahemmet University, Stockholm, Sweden; 2 Department of Neurobiology, Care Sciences and Society, Division of Physiotherapy, Karolinska Institutet, Stockholm, Sweden; 3 Academic Primary Care Center, Region Stockholm, Stockholm, Sweden; 4 Department of Nutrition, Dietetics and Food Studies, Uppsala University, Uppsala, Sweden; 5 School of Health Sciences, Örebro University, Örebro, Sweden; 6 Department of Molecular Medicine and Surgery, Karolinska Institutet, Stockholm, Sweden; 7 Rolf Luft Research Center for Diabetes and Endocrinology, Karolinska University Hospital, Stockholm, Sweden; 8 School of Kinesiology, Shanghai University of Sport, Shanghai, China; 9 College of Health Solutions, Arizona State University, Phoenix, AZ USA; 10 Smedby Primary Care Center, Kalmar, Sweden; 11 Department of Clinical Science and Education, Södersjukhuset, Karolinska Institutet, Stockholm, Sweden

**Keywords:** Activity tracker, Behaviour, Cardiometabolic risk factors, HbA1c, Pedometer, Physical activity, Step counter

## Abstract

**Background:**

This aimed to evaluate the effects of self-monitoring of daily steps with or without counselling support on HbA1c, other cardiometabolic risk factors and objectively measured physical activity (PA) during a 2-year intervention in a population with prediabetes or type 2 diabetes.

**Methods:**

The Sophia Step Study was a three-armed parallel randomised controlled trial. Participants with prediabetes or type 2 diabetes were recruited in a primary care setting. Allocation (1:1:1) was made to a multi-component intervention (self-monitoring of steps with counselling support), a single-component intervention (self-monitoring of steps without counselling support) or standard care. Data were collected for primary outcome HbA1c at baseline and month 6, 12, 18 and 24. Physical activity was assessed as an intermediate outcome by accelerometer (ActiGraph GT1M) for 1 week at baseline and the 6-, 12-, 18- and 24-month follow-up visits. The intervention effects were evaluated by a robust linear mixed model.

**Results:**

In total, 188 subjects (64, 59, 65 in each group) were included. The mean (SD) age was 64 (7.7) years, BMI was 30.0 (4.4) kg/m^2^ and HbA1c was 50 (11) mmol/mol, 21% had prediabetes and 40% were female. The dropout rate was 11% at 24 months. Effect size (CI) for the primary outcome (HbA1c) ranged from -1.3 (-4.8 to 2.2) to 1.1 (-2.4 to 4.6) mmol/mol for the multi-component vs control group and from 0.3 (-3.3 to 3.9) to 3.1 (-0.5 to 6.7) mmol/mol for the single-component vs control group. Effect size (CI) for moderate-to-vigorous physical activity ranged from 8.0 (0.4 to 15.7) to 11.1 (3.3 to 19.0) min/day for the multi-component vs control group and from 7.6 (-0.4 to 15.6) to 9.4 (1.4 to 17.4) min/day for the single-component group vs control group.

**Conclusion:**

This 2-year intervention, including self-monitoring of steps with or without counselling, prevented a decrease in PA but did not provide evidence for improved metabolic control and cardiometabolic risk factors in a population with prediabetes or type 2 diabetes.

**Trial registration:**

ClinicalTrials.gov, NCT02374788. Registered 2 March 2015—Retrospectively registered.

**Supplementary Information:**

The online version contains supplementary material available at 10.1186/s12966-021-01193-w.

## Background

For individuals with or at risk of type 2 diabetes, metabolic control is of utmost importance to reduce the risk of cardiovascular disease and other complications [1]. Aerobic exercise improves metabolic function and reduces cardiovascular risk factors in individuals with prediabetes or type 2 diabetes [2]. Regular physical activity (PA) is therefore central in diabetes self-management and prevention, and health care providers are encouraged to give support for PA in individuals with prediabetes or type 2 diabetes [1]. Yet, most people with prediabetes or type 2 diabetes do not reach the recommended levels of PA [3, 4].

Walking is an activity that most people can perform and is a useful therapeutic tool for individuals with type 2 diabetes [5]. Step counting is a measure of locomotor movement (e.g., walking) and daily steps correlate well with both total PA and moderate-to-vigorous PA (MVPA) [6]. Several systematic reviews and meta-analyses show that self-monitoring of PA (e.g., step counting) as a motivational tool has positive short-term effects on PA levels [7–13] and weight [8, 14] in people with type 2 diabetes. The use of step counters is recommended to encourage and maintain PA [15, 16]. However, evidence for the effect of self-monitoring of steps on metabolic control and cardiovascular risk factors is inconsistent [7, 9–11, 14].

There is evidence for stronger PA effects with resource-demanding counselling support than minimal interventions, including using step counters only [10]. However, the evidence is conflicting [9, 11, 16]. Moreover, the effectiveness of long-term (> 12 months) interventions implemented in a primary care setting remains unclear [10, 12, 17].

The Sophia Step Study was undertaken as a three-armed randomised controlled trial (RCT) to evaluate self-monitoring of daily steps with and without counselling in individuals with prediabetes or type 2 diabetes in a primary care context [18]. The study hypothesised that both intervention groups would increase PA levels and subsequently improve metabolic control and reduce cardiovascular risk factors. We also hypothesied that the multi-component group (including counselling) would maintain the effects at a higher level over time than the single-component intervention group (without counselling). The primary outcome was HbA1c as a measure of metabolic control. Secondary outcomes included several clinical and anthropometric measures of cardiovascular risk. Objectively measured PA (MVPA, light-intensity PA (LPA), time spent in sedentary behaviours (SB) and daily steps) were applied as intermediate outcomes. The intervention duration was 2 years.

## Methods

### Aim

This study aimed to evaluate the effects of self-monitoring of steps with or without counselling support on HbA1c, other cardiometabolic risk factors and PA during a 24-month intervention in individuals with prediabetes or type 2 diabetes.

### Study design, setting and materials

The study was a three-armed parallel randomised RCT. It was performed according to the CONSORT guidelines for reporting non-pharmacological treatment interventions and multi-arm parallel group randomised trials. Patients at two urban and one rural primary care centre were recruited by their diabetes specialist nurse. Eight rounds of recruitments were made, varying across seasons between April 2013 and January 2018. Some 385 persons were invited to enrol in the study during regular visits to the primary care centre or by mailed invitation. Before baseline measurements, patients were examined by a general practitioner to ensure study eligibility. Inclusion and exclusion criteria are listed in Table 1. Participants were randomly assigned to a multi-component intervention group (self-monitoring of steps with counselling support), a single-component intervention group (self-monitoring of steps without counselling support) or a control group (standard care). Randomization was conducted using sealed envelopes prepared by project staff and distributed by the diabetes specialist nurses, stratified by gender, at an allocation ratio of 1:1:1. Demographics were collected by a questionnaire at baseline, web- or paper-based as the participant preferred. Data on health conditions and medications were obtained from medical records and asked for at the baseline assessment. Specific details on methods are published in a study protocol [18].

**Table 1:** Inclusion and exclusion criteria

**Inclusion criteria**	**Exclusion criteria**
- 40–80 years - Prediabetes (HbA_1c_ > 39- < 47 mmol/mol and/or fasting glucose > 5.6 mmol/l) or diagnosed with type 2 diabetes with a duration of ≥ 1 year - Ability to communicate in Swedish	- Myocardial infarction in the past 6 months - Serum creatinine > 140 mmol/l - Diabetic foot ulcer or risk of ulcer (severe peripheral neuropathy) - On insulin for the past 6 months - Additional disease prohibiting physical activity - Repeated hypoglycaemia or severe hypoglycaemia in the past 12 months - Being very physically active according to the Stanford Brief Activity Survey [19] - Having no access to the internet

### Interventions

Details and theoretical framework of the 2-year intervention are described in the study protocol [18]. The fidelity of the intervention is outlined in a process evaluation [20]. Participants in the two intervention groups were offered step counters (Yamax Digiwalker SW 200: Yamax Corporation, Tokyo, Japan) and directed to a website for self-monitoring of steps [21]. The participants of the multi-component group were, in addition to pedometers and the website, offered 12 group consultations (10 during the first year) and nine individual face-to-face consultations (seven during the first year) by their diabetes specialist nurse. The group sessions were led by project staff (the urban centres) and a diabetes specialist nurse (rural centre). The programme for the group sessions was guided by the health belief model [22], social cognitive theory [23] and the transtheoretical model of change [24]. The programme considered several techniques for behaviour change [18]. Individual consultations were based on a motivational interviewing technique [25]. Standard care included meeting a diabetes specialist nurse and a physician once a year, or more often if needed. All participants were offered study assessments at baseline and at follow-up (2, 3, 4, 6, 9, 12, 18 and 24 months), including feedback on health outcomes. The multi-component group had 45 min with their diabetes nurse consisting of counselling and study assessments. The single-component and control group had 15 min with their diabetes nurse for study assessment.

### Outcome measures

#### Cardiometabolic risk factors

Data on biomarkers collected at baseline and at the 6-, 12-, 18- and 24-month follow-up were used for study analysis. Biomarkers included HbA_1c_ (mmol/mol), fasting blood glucose (mmol/l), triglycerides (mmol/l), high-density lipoprotein cholesterol (HDL) (mmol/l), low-density lipoprotein cholesterol (LDL) (mmol/l), ApoB/ApoA1 (g/l) and C-peptide (nmol/l). Data collected at baseline and follow-up (6, 12, 18 and 24 months) were used for resting systolic blood pressure (mmHg), resting diastolic blood pressure (mmHg) and anthropometric variables: weight (kg), body fat (%), waist circumference (cm) and sagittal abdominal diameter (cm). Blood samples were analysed by standardised methods at the Research laboratory, Karolinska Hospital according to the manufacturer’s kit test instructions. HbA1c was determined using a Variant II Turbo HbA1c analyser (Bio-Rad Laboratories, USA). Plasma glucose, triglycerides, total cholesterol, HDL, LDL, Apolipoprotein-A1 and Apolipoprotein B and C-peptide were determined using analysers from Beckman Coulter, Inc. USA and Roche Diagnostics, Switzerland. Detailed descriptions of measuring and analysis methods are provided elsewhere [18].

#### Physical activity and sedentary behaviours

PA spent in MVPA and LPA, SB and daily steps were measured for 1 week at baseline and the 6-, 12-, 18- and 24-month follow-up with a hip-worn ActiGraph GT1M accelerometer (ActiGraph, Pensacola, FL). MVPA was chosen as the main physical activity outcome [2]. Data collection and data processing procedures have been published previously [26]. Non-wear time was determined at 90 min with consecutive zero counts, allowing for 2 min interval of nonzero counts [27]. Participants providing data of ≥ 10 h per day for at least 3 days were included in the analyses [28]. Commonly applied count-based thresholds of SB < 100 counts per minute (cpm) [29], LPA (100–1951 cpm) and MVPA (≥ 1952 cpm) [30] were applied.

### Statistical analyses

Statistical analyses were performed using the R statistical software (version 4.0.3) by an external statistician blinded to group allocation. The data were examined for normality, outliers and missing values. Change from baseline to the follow-up visits was examined for each randomisation group by computing the mean and 95% confidence interval for the participants’ difference in follow-up value and baseline values.

The between group comparisons were performed with an intent-to-treat approach using linear mixed model including subject as a random effect (random intercept) and age, randomization group, time as well as a randomization group and time interaction as fixed factors [31]. Due to deviations from the normality assumption for the residuals, a robust variant of the linear mixed model was fitted. The robust linear mixed model applies weighting to the observations by giving less weight to individuals with large deviations from normality [32]. Analyses of SB were adjusted for wear time. Sensitivity analysis were conducted for participants with type 2 diabetes (excluding prediabetes participants). The study was designed to have 80% power (alpha = 0.05) to detect a difference of 4 mmol/mol (0.6%) in HbA1c between the groups at 12 months, revealing that we required a sample size of at least 56 participants in each group.

## Results

**Fig. 1:**
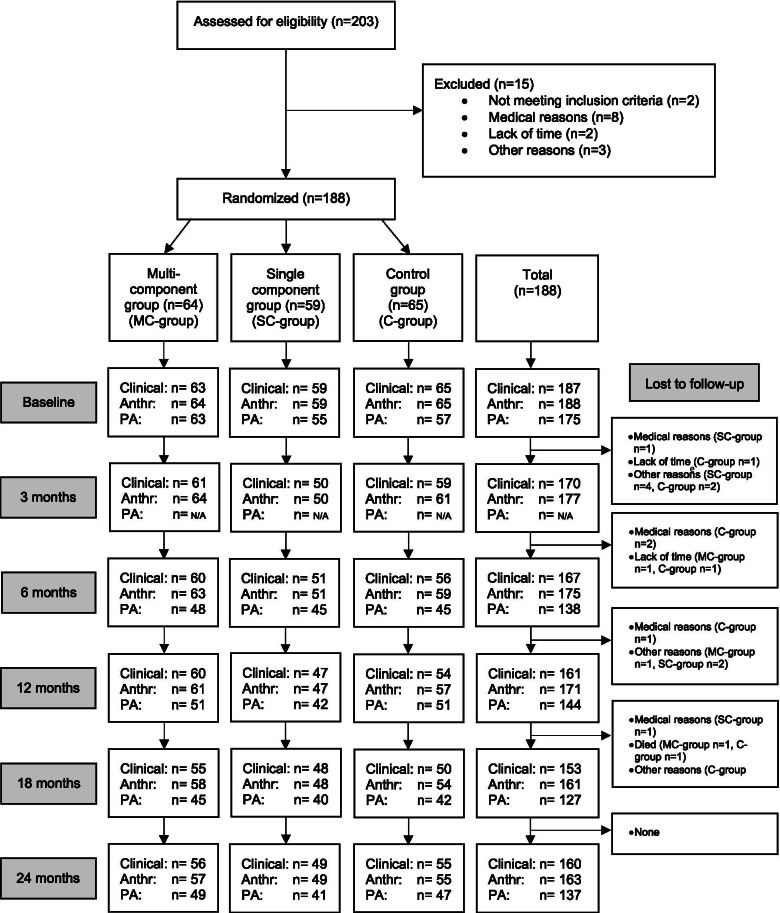
Flowchart of enrollment and participation in the Sophia Step Study. Clinical presents number participants with valid data for HbA1c, Anthr = Anthropometrics presents number participants with valid data for weight, PA = Physical activity presents number participants with valid data for moderate-to-vigorous physical activity

Of the 385 invited patients, 203 (53%) agreed to participate. By November 2018, 188 patients from three primary care centres fulfilled the inclusion criteria and were randomised into one of the intervention groups or the control group. The dropout rate was 9% at 12 months and 11% at 24 months. The response rate of invited and eligible individuals was 49%. A process evaluation with details on the context, reasons for declining and adherence to the intervention has been published elsewhere [20]. Figure 1 depicts the number of participants recruited, excluded, declined, consented and dropouts per intervention group, as well as reasons for exclusion and termination.

Table 2 describes participant baseline characteristics by allocated group. The mean age in all groups was 64 ± 7.7 years, 21% had prediabetes, 40% were female, 72% lived with a partner, 51% had a university education, mean BMI was 30.0 ± 4.4 and mean HbA1c was 50 ± 11.4 mmol/mol. Additional file 1 provides characteristics by the completers and dropouts and Additional file 2 provides baseline characteristics by diagnosis (prediabetes or type 2 diabetes). Medications and onset of diseases during the interventions in all three groups are described in Additional file 3. No adverse health events due to participation were reported.

**Table 2:** Baseline characteristics by intervention group

	**Total (*n* = 188)**	**Multi-component intervention (*n* = 64)**	**Single-component intervention (*n* = 59)**	**Control group (*n* = 65)**
**Demographics**				
Age (years), mean (SD)	64.1 (7.7)	64.2 (6.9)	65.1 (7.3)	63.1 (8.7)
Female, n (%)	76 (40.4)	28 (43.8)	24 (40.7)	24 (36.9)
Prediabetes, n (%)	40 (21.2)	13 (20.3)	10 (16.9)	17 (26.2)
Diabetes duration in years, mean (SD)^a^	8.2 (5.9)	9.4 (7.1)	7.9 (5.2)	7.3 (4.8)
Daily smoker, n (%)^b^	12 (7.1)	3 (5.1)	4 (7.7)	5 (8.6)
University education, n (%)^b,c^	87 (50.6)	28 (45.9)	25 (47.2)	34 (58.6)
Living with a partner, n (%)^b^	124 (72.1)	46 (75.4)	37 (71.2)	41 (69.5)
Start of the intervention				
During autumn, n (%)	98 (52.1)	33 (51.6)	33 (55.9)	32 (49.2)
During winter, n (%)	70 (37.2)	22 (34.4)	24 (40.7)	24 (36.9)
During spring, n (%)	19 (10.1)	9 (14.1)	1 (1.7)	9 (13.8)
During summer, n (%)	1 (0.5)	0 (0)	1 (1.7)	0 (0)
**Cardiometabolic risk factors**				
HbA1c (mmol/mol), mean (SD)	49.9 (11.4)	49.4 (11.2)	50.5 (11.2)	49.9 (12.0)
Fasting glucose (mmol/L), mean (SD)	7.9 (1.9)	7.7 (1.8)	8.0 (2.2)	7.8 (1.8)
C-peptide (nmol/L), mean (SD)	1.07 (0.44)	1.12 (0.46)	1.00 (0.49)	1.09 (0.36)
Insulin (mU/l), mean (SD)	14.8 (9.6)	15.7 (9.6)	13.6 (10.7)	14.9 (8.7)
ApoB/ApoA1, mean (SD)	0.7 (0.2)	0.7 (0.2)	0.6 (0.2)	0.8 (0.2)
HDL cholesterol (mmol/L), mean (SD)	1.4 (0.4)	1.4 (0.3)	1.3 (0.4)	1.4 (0.5)
LDL cholesterol (mmol/L), mean (SD)	2.9 (1.0)	2.9 (0.9)	2.8 (1.0)	3.0 (1.0)
Total cholesterol, (mmol/L) mean (SD)	5.01 (1.08)	5.03 (0.98)	4.90 (1.11)	5.09 (1.14)
Triglycerides (mmol/L), mean (SD)	1.74 (1.01)	1.80 (0.87)	1.78 (1.16)	1.65 (1.00)
Body mass index (kg/m^2^), mean (SD)	30.0 (4.4)	30.3 (4.1)	29.4 (4.4)	30.2 (4.8)
Body fat (%), mean (SD)	34.7 (8.1)	36.0 (8.2)	33.7 (7.8)	34.1 (8.1)
Waist circumference, men (cm), mean (SD)	107.2 (10.6)	107.0 (10.8)	107.4 (11.5)	107.1 (9.7)
Waist circumference, women (cm), mean (SD)	99.3 (12.3)	101.9 (11.9)	95.3 (11.8)	100.1 (12.8)
Sagittal abdominal diameter (cm), mean (SD)	24.7 (3.6)	24.8 (3.4)	24.6 (4.0)	24.7 (3.5)
Systolic blood pressure (mmHg), mean (SD)	134.3 (15.9)	131.2 (14.4)	138.2 (16.1)	133.9 (16.4)
Diastolic blood pressure (mmHg), mean (SD)	83.8 (9.2)	83.1 (7.9)	84.8 (9.4)	83.5 (10.1)
**Physical activity and sedentary behaviours**				
MVPA (min/day), mean (SD)^e^	29.3 (23.7)	28.9 (20.5)	29.6 (25.0)	29.6 (25.9)
LPA (min/day), mean (SD)^e^	220.0 (65.4)	213.3 (59.7)	222.5 (72.3)	225.2 (64.9)
SB (min/day), mean (SD)^e^	588.5 (84.9)	596.6 (89.6)	583.5 (91.1)	584.3 (73.3)
Steps/day, mean (SD)^d^	6570 (3090)	6613 (3305)	6559 (3125)	6540 (2887)
Accelerometer wear time (min/day), mean (SD)^e^	837.9 (74.1)	838.8 (92.3)	835.7 (59.6)	839.1 (65.0)
Met PA recommendation of > 150 min MVPA/week, n (%)^e^	94 (53.7)	39 (61.9)	29 (52.7)	26 (45.6)

The table presents the mean (standard deviation) or proportion (%). The number of participants varies with ± 1–2 for some variables due to missing data

*MVPA* Moderate-to-vigorous intensity physical activity, *LPA* Time in light-intensity physical activity, *SB* Time in sedentary behaviours, *min* Minutes

^a^Only participants diagnosed with diabetes

^b^Some participants have missing data

^c^Having university education

^d^Baseline daily steps were available for 165 participants

^e^Baseline accelerometry data were available for 177 participants

**Fig. 2:**
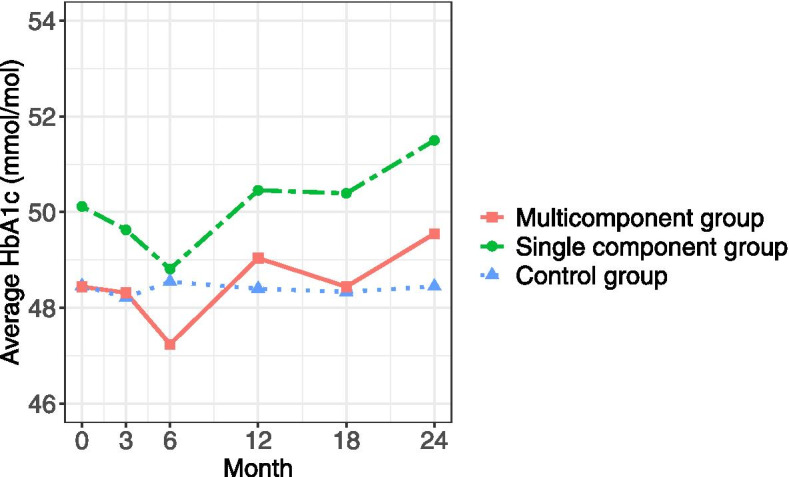
Changes in HbA1c over time. Values are based on predicted group means from the linear mixed model analysis

**Fig. 3:**
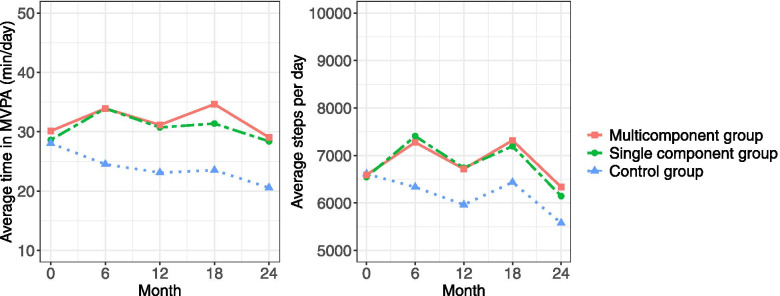
Changes in physical activity over time. MVPA = Time in moderate-to-vigorous physical activity. Values are based on predicted group means from the linear mixed model analysis

Mean change in HbA1c, MVPA and daily steps for each group over 24 months are illustrated in Figs. 2 and 3. Additional files 4 and 5 show the mean change in HbA1c, MVPA and daily steps for each group over 24 months with confidence intervals (CI). Additional file 6 reports mean (CI) within-group differences between baseline and each follow-up. There is a trend towards improved HbA1c values in both intervention groups at 6 months, which is not seen in the control group. After 12 months, the improvement in HbA1c had reversed and at 24 months exceeded baseline levels. Mean MVPA and daily steps increased during the first 6 months for both intervention groups and showed a mixed pattern of improvement-maintenance during the intervention period. The control group decreased mean levels of MVPA and daily steps during the intervention period.

The intervention effects (between-group differences) for HbA1c, MVPA, LPA, SB and daily steps are displayed in Tables 3 and 4. There was no intervention effect on any of the cardiometabolic variables (Additional file 7).

**Table 3:** Intervention effect over time between the two intervention groups and control group on HbA1c

	**Intervention effect at 3 months (95% CI)**	**Intervention effect at 6 months (95% CI)**	**Intervention effect at 12 months (95% CI)**	**Intervention effect at 18 months (95% CI)**	**Intervention effect at 24 months (95% CI)**
**Multi-component intervention vs. control group**					
HbA1c (mmol/mol)	0.1 (-3.4 to 3.6)	-1.3 (-4.8 to 2.2)	0.6 (-2.9 to 4.1)	0.1 (-3.4 to 3.6)	1.1 (-2.4 to 4.6)
**Single-component intervention vs. control group**					
HbA1c (mmol/mol)	1.4 (-2.2 to 5.0)	0.3 (-3.3 to 3.9)	2.1 (-1.5 to 5.7)	2.1 (-1.6 to 5.7)	3.1 (-0.5 to 6.7)

Effect sizes and 95% confidence interval (CI) from the robust repeated linear mixed model analysis

**Table 4:** Intervention effect over time between the two interventions and control group on physical activity and sedentary behaviour

	**Intervention effect at 6 months (95% CI)**	**Intervention effect at 12 months (95% CI)**	**Intervention effect at 18 months (95% CI)**	**Intervention effect at 24 months (95% CI)**
**Multi-component intervention vs. control group**				
MVPA (min/day)	9.4 (1.6 to 17.2)	8.0 (0.4 to 15.7)	11.1 (3.3 to 19.0)	8.5 (0.8 to 16.2)
LPA (min/day)	-11.7 (-37.3 to 14.0)	-8.1 (-33.3 to 17.1)	-12.8 (-38.8 to 13.1)	-1.4 (-26.9 to 24.1)
SB, adjusted for wear time, (min/day)	3.6 (-23.5 to 30.7)	-3.5 (-30.3 to 23.2)	-4.5 (-31.9 to 22.9)	-4.1 (-31.1 to 22.8)
Daily steps	943 (-147 to 2033)	754 (-318 to 1826)	881 (-219 to 1983)	758 (-324 to 1840)
**Single-component intervention vs. control group**				
MVPA (min/day)	9.4 (1.4 to 17.4)	7.6 (-0.4 to 15.6)	7.9 (-0.3 to 16.0)	7.8 (-0.2 to 15.9)
LPA (min/day)	5.5 (-21.0 to 31.9)	1.3 (-25.1 to 27.6)	-6.0 (-32.9 to 20.9)	-10.3 (-36.9 to 16.3)
SB, adjusted for wear time, (min/day)	-14.9 (-42.8 to 13.1)	-12.1 (-40.0 to 15.7)	-9.5 (-37.8 to 18.9)	1.6 (-26.4 to 29.7)
Daily steps	1074 (-50 to 2197)	778 (-340 to 1896)	760 (-380 to 1900)	567 (-560 to 1694)

Effect sizes and 95% confidence interval (CI) for physical activity and sedentary behaviours from the robust repeated linear mixed model analysis

*MVPA* Moderate-to-vigorous intensity physical activity, *LPA* Time in light-intensity physical activity, *SB* Time in sedentary behaviours, *min* Minutes

For the PA variables, the two intervention groups showed a comparable pattern over time, with improved levels at a similar range compared to the control group. Some variations occurred in the increase in PA, reaching statistically significant levels only for MVPA in the multi-component intervention group versus the control group. The result from the sensitivity analyses, including only participants with type 2 diabetes, did not differ from the main result, except for a more pronounced intervention effect on daily steps at 6 months for both intervention groups. The intervention was delivered as intended, and no unintended effects were reported.

## Discussion

This paper describes the results of a 2-year, three-armed RCT conducted in a primary health care context. The study sought to evaluate self-monitoring of daily steps with (multi-component) and without (single-component) counselling versus a control group in individuals with prediabetes or type 2 diabetes on clinical, anthropometric and behaviour outcomes. The hypothesis tested was that both intervention groups would increase PA levels and subsequently improve metabolic control and reduce cardiovascular risk factors compared to the control group, and the multi-component group would maintain the effects ata higher level over time than the single-component group.

The main finding is that this study does not provide evidence of an intervention effect in either intervention group on the primary outcome (HbA1c) or the other measured cardiometabolic risk factors. Both intervention groups showed tendencies for beneficial changes in HbA1c at 6 months, but the distribution of improvement was large, and the findings did not reach clinically relevant levels for most individuals. The favourable changes in HbA1c returned to baseline levels by month 12 and exceeded baseline levels at 24 months. The initial beneficial change in HbA1c can be explained by the increase in PA shown at 6 months when the intervention was most intense for the intervention components. However, it is unknown whether the changes in PA and HbA1c were causally linked to the intervention. To establish such a relationship subgroup analyses are needed. Yet, another explanation for the initial beneficial change is that the participants were highly motivated for behaviour change at the start of the intervention but failed to maintain the motivation for long-term behaviour change. To uphold metabolic control is known to be challenging due to the progressive nature of the disease, even with improvement in the quality of diabetes care [33].

The lack of evidence for an effect on metabolic control and cardiometabolic risk factors in this study adds to the body of conflicting evidence documenting the effect of step counters on metabolic control and cardiovascular risk factors [7, 9–11, 14]. The study indicates that step counters may be ineffective in improving metabolic control. However, with regard to the levels of PA and HbA1c, some clarifications can be made. The high level of baseline PA in this sample may explain the rather beneficial baseline clinical values, reaching a ceiling effect for many individuals. A recent meta-analysis suggests that every 1000 steps/day that individuals take compared to reference individuals reduces the risk of cardiovascular morbidity or mortality by 5–21% [34]. An intervention of long duration should consider trends and natural changes made in society. A comparison can be made to trends in metabolic control occurring during the intervention period that reflect improvements in diabetes care. The National Diabetes Registry data show that mean HbA1c in the general Swedish type 2 diabetes population decreased from 54.1 mmol/mol in 2015 to 52.8 mmol/mol in 2020 [35].

Due to multiple testing, interpretations of the results should be made with care. Still, there is a tendency towards an intervention effect on the maintenance of PA in both intervention groups, slightly favouring the multi-component intervention. The statistically significant effect on PA was robust over time for MVPA. The effect size (mean difference of MVPA in the multi-component intervention group compared to the control group) was 8–11 min across the 2 years follow-up. Daily steps show the same pattern as MVPA, with a variation of 754–943 steps/day between the multi-component intervention group and the control group over the 2 years but did not reach statistically significant levels. The effect of the single-component intervention did not reach statistically significant levels but followed the same pattern as the multi-component intervention (8–9 min/day for MVPA and 567–1074 for steps/day). The between-group differences are mainly explained by the intervention groups maintaining mean PA levels over the 2 years, while the control group decreased the mean PA levels and increased mean sedentary time.

While not part of the hypotheses tested, the mean level of increase in PA by the intervention groups varied over the 2 years. The increase after 6 months (876 steps/day in the multi-component intervention group and 1009 steps/day in the single-component intervention group) was somewhat lower than that found in previous similar studies using step counters that range from 1281 to 2744 steps/day [36–38]. The increase after 12 months (89 steps/day in the multi-component and 272 steps/day in the single-component intervention group) corresponds to an increase of 91 to 1220 steps/day [38–42] in previous 12-month interventions. An increase of 500 steps/day or a 5–6-min walk/day is a clinically meaningful increase that can reduce cardiovascular morbidity and mortality in inactive individuals [43]. Note that the control group reduced the mean level of PA by nearly 1000 steps/day and nearly 10 min/day of MVPA from the baseline period to month 24. While the intervention groups had a small decrease in the number of daily steps taken from baseline to 24 months, both groups maintained their minutes of MVPA at month 24 compared to baseline values. These differences between interventions and control group underline the importance of providing support for PA in this adult population with prediabetes or type 2 diabetes. While this study does not provide sufficient evidence for intervention effects on metabolic control, self-monitoring of steps may still be an acceptable, low-cost, low-risk approach to improving PA.

There was no benefit from the multi-component intervention in this study compared to the single-component intervention. Studies with comparable interventions and populations show somewhat conflicting findings, as do two recent meta-analyses with broader populations [10, 16]. In the PACE-UP trial the group receiving pedometers only increased daily steps to the same level as the group receiving pedometers and three counselling sessions [41]. Although of varying lengths and support intensity, other similar trials reported superior effects in intervention groups receiving step counting and counselling versus step counting alone [44–46].

A process evaluation of the Sophia Step Study has been undertaken, providing details of the intervention delivery and context, as well as evidence for feasibility and a high degree of reach of the interventions [20]. Thus, the lack of intervention effect in this study cannot be explained by unsuccessful intervention delivery. However, the study sample was small (from 59–64 per group) and both baseline PA levels and PA changes varied appreciably in the study participants. Secondary analyses are planned to analyse PA patterns over time and responsiveness in metabolic control and cardiovascular risk of subgroups based on initial PA levels and changes in PA. Further analyses are also planned to assess the effect of the intervention on health-related quality of life and aspects of mental health.

The main strength of this study is the long intervention duration and repeated measures of both objectively measured PA and variation of cardiometabolic risk factor. The randomisation was successful with a between-group variation at baseline for waist circumference among women and systolic blood pressure only. Linear mixed model analyses included baseline values and between-group differences were thus accounted for. Both genders were included, representing the Swedish diabetes population with a majority of men. Recruitment was conducted during autumn, winter and spring to reduce seasonal variations in physical activity.

The study has some limitations. We aimed to target people with low levels of PA but apparently also reached individuals that were already sufficiently active in accordance with the recommended level of a minimum of 150 min of MVPA per week. The accelerometer used has limitations as it is not sensitive enough to detect certain activities (e.g., swimming and bicycling). However, the likelihood is low that the detection limit of the accelerometer may have affected the effect on PA between the groups. Although the Sophia Step Study was an RCT, internal validation could have been violated. The repeated study assessments for all three groups may have influenced motivation to comply with a healthy lifestyle and medication regimen in the intervention groups and among the control participants. The same diabetes specialist nurse met all participants, regardless of intervention allocation. The nurses were trained in motivational interviewing and we cannot rule out that there was a diffusion effect among the intervention groups. The dropouts had a slightly detrimental health status and less time in MVPA at baseline and might have benefited the most from increasing PA. Power was calculated based on the primary outcome, HbA1c, and we did not fully reach the intended sample size. There is thus a risk of a type 2 error when interpreting the findings. The study includes individuals with prediabetes and type 2 diabetes and was underpowered to detect an effect on HbA1c on the diagnostic groups separately. Finally, the study covered three primary care centres with slightly different catchment areas. However, the educational level of the sample is quite high and therefore the results may not generalise to all populations and settings.

## Conclusion

This 2-year intervention, including self-monitoring of steps with or without counselling, prevented a decrease in PA but did not provide evidence for improved metabolic control and cardiometabolic risk factors in a population with prediabetes or type 2 diabetes.

## Supplementary Information

**Additional file 1: Table.** Description of dropouts.

**Additional file 2: Table.** Description per diagnose (prediabetes and type 2 diabetes).

**Additional file 3: Table.** Description of changes in medications.

**Additional file 4: Figure.** Mean change in HbA1c for each group over 24 months with confidence intervals included.

**Additional file 5: Figure.** Mean change in MVPA and daily steps for each group over 24 months with confidence intervals included.

**Additional file 6: Table.** Description of changes in each group.

**Additional file 7: Table.** Effects on secondary outcomes.

## Data Availability

The datasets generated or analysed during the current study are not publicly available because data can be traced back to the study participants. According to Swedish and EU data legislation, access can only be granted upon a reasonable request. The request should be addressed to the PI and will be handled on a case-by-case basis. Any sharing of data will be regulated via a data transfer and use agreement with the recipient.
