# Supplementary material for: Effects of a three-armed randomised controlled trial using self-monitoring of daily steps with and without counselling in prediabetes and type 2 diabetes—the Sophia Step Study
Source: Int J Behav Nutr Phys Act. 2021 Sep 8;18:121. doi: 10.1186/s12966-021-01193-w (PMC8424865; doi:10.1186/s12966-021-01193-w)
Supplement: Supplementary file 1 — Additional file 1: Table. Description of dropouts. [file 12966_2021_1193_MOESM1_ESM.docx]

Table S1. Descriptives divided by completers and drop-outs.

|  | **Total**  **(n=188)** | **Completers**  **(n=167)** | **Drop-outs**  **(n=21)** |
| --- | --- | --- | --- |
| **Demographics** |  |  |  |
| Age (years), mean (SD) | 64.1 (7.7) | 64.4 (7.6) | 61.8 (8.6) |
| Female, n (%) | 76 (40.4) | 68 (40.7) | 8 (38.1) |
| Daily smoker, n (%) | 12 (7.1) | 9 (6.0) | 3 (16.7) |
| University education, n (%) | 87 (51) | 74 (48.1) | 13 (72.2) |
| Living with partner, n (%) | 124 (72) | 111 (66.5) | 13 (72.2) |
| **Cardiometabolic risk factors** |  |  |  |
| HbA1c (mmol/L), mean (SD) | 49.9 (11.4) | 49.5 (11.5) | 53.3 (10.1) |
| Fasting glucose (mmol/L), mean (SD) | 7.9 (1.9) | 7.8 (1.9) | 8.4 (1.9) |
| C-Peptide (nmol/L), mean (SD) | 1.1 (0.4) | 1.1 (0.5) | 1.1 (0.3) |
| ApoB/ApoA1, mean (SD) | 0.7 (0.2) | 0.7 (0.2) | 0.8 (0.2) |
| HDL cholesterol (mmol/L), mean (SD) | 1.4 (0.4) | 1.4 (0.4) | 1.2 (0.3) |
| LDL cholesterol (mmol/L), mean (SD) | 2.9 (1.0) | 2.8 (1.0) | 3.3 (0.9) |
| Total cholesterol, mean (SD) | 5.0 (1.1) | 5.0 (1.1) | 5.3 (1.0) |
| Triglycerides (mmol/L), mean (SD) | 1.7 (1.0) | 1.7 (1.0) | 1.9 (1.0) |
| Body Mass Index (kg/m^2^), mean (SD) | 30.0 (4.4) | 29.9 (4.3) | 30.3 (5.5) |
| Body fat, mean (SD) | 34.7 (8.1) | 34.8 (7.9) | 33.4 (9.6) |
| Waist circumference men (cm), mean (SD) | 107.1 (10.5) | 107.3 (10.6) | 106.6 /10.3) |
| Waist circumference women (cm), mean (SD) | 99.3 (12.3) | 98.7 (12.2) | 104.3 (12.6) |
| Sagittal abdominal diameter (cm), mean (SD) | 24.7 (3.6) | 24.7 (3.6) | 24.9 (3.4) |
| Systolic blood pressure (mmHg), mean (SD) | 134.3 (15.9) | 134.7 (15.6) | 131.2 (18.1) |
| Diastolic blood pressure (mmHg), mean (SD) | 83.8 (9.2) | 83.9 (9.0) | 82.6 (10.5) |
| **Physical activity and sedentary behaviour** |  |  |  |
| Steps/day, mean (SD) | 6570 (3090) | 6699 (3054) | 5467 (3273) |
| MVPA (min/day), mean (SD)^5^ | 29.3 (23.7) | 29.4 (22.6) | 20.6 (23.8) |
| LPA (min/day), mean (SD)^5^ | 220.1 (65.4) | 222.1 (64.4) | 200.9 (72.6) |
| SB (min/day), mean (SD)^5^ | 588.5 (84.9) | 587.1 (84.2) | 600.9 (91.7) |
| Reach PA recommendation of >150 min MVPA/week, n (%) | 94 (53.7) | 88 (56.1) | 6 (33.3) |
