# Supplementary material for: Effects of a three-armed randomised controlled trial using self-monitoring of daily steps with and without counselling in prediabetes and type 2 diabetes—the Sophia Step Study
Source: Int J Behav Nutr Phys Act. 2021 Sep 8;18:121. doi: 10.1186/s12966-021-01193-w (PMC8424865; doi:10.1186/s12966-021-01193-w)
Supplement: Supplementary file 2 — Additional file 2: Table. Description per diagnose (prediabetes and type 2 diabetes). [file 12966_2021_1193_MOESM2_ESM.docx]

Table S1. Descriptives divided by diagnose: type 2 diabetes or prediabetes

|  | **Total**  **(n=188)** | **Type 2 diabetes**  **(n=148)** | **Prediabetes**  **(n=40)** |
| --- | --- | --- | --- |
| **Demographics** |  |  |  |
| Age (years), mean (SD) | 64.1 (7.7) | 63.8 (7.9) | 65.1 (7.0) |
| Female, n (%) | 76 (40.4) | 52 (35) | 24 (60) |
| Diagnose duration (years), mean (SD) | N/A | 8.2 (5.9) | 1.9 (1.8) |
| Daily smoker, n (%) | 12 (7.1) | 10 (7.4) | 2 (5.9) |
| University education, n (%) | 87 (51) | 65 (48) | 22 (60) |
| Living with partner, n (%) | 124 (72) | 101 (75) | 23 (62) |
| **Cardiometabolic risk factors** |  |  |  |
| HbA1c (mmol/L), mean (SD) | 49.9 (11.4) | 52.7 (11.2) | 39.8 (4.1) |
| Fasting glucose (mmol/L), mean (SD) | 7.9 (1.9) | 8.3 (2.0) | 6.4 (0.7) |
| C-Peptide (nmol/L), mean (SD) | 1.1 (0.4) | 1.1 (0.5) | 1.0 (0.3) |
| ApoB/ApoA1, mean (SD) | 0.7 (0.2) | 0.7 (0.2) | 0.7 (0.2) |
| HDL cholesterol (mmol/L), mean (SD) | 1.4 (0.4) | 1.3 (0.4) | 1.6 (0.4) |
| LDL cholesterol (mmol/L), mean (SD) | 2.9 (1.0) | 2.8 (1.0) | 3.3 (0.8) |
| Total cholesterol, mean (SD) | 5.01 (1.08) | 4.9 (0.0) | 5.6 (0.8) |
| Triglycerides (mmol/L), mean (SD) | 1.7 (1.0) | 1.8 (1.1) | 1.5 (0.8) |
| Body Mass Index (kg/m^2^), mean (SD) | 30.0 (4.4) | 30.1 (4.6) | 29.7 (3.9) |
| Body fat, mean (SD) | 34.65 (8.05) | 34.1 (8.0) | 36.8 (7.8) |
| Waist circumference men (cm), mean (SD) | 107.1 (10.5) | 107.2 (10.3) | 107.3 (12.1) |
| Waist circumference women (cm), mean (SD) | 99.3 (12.3) | 100.6 (12.9) | 96.4 (10.7) |
| Sagittal abdominal diameter (cm), mean (SD) | 24.71 (3.60) | 25.0 (3.5) | 23.4 (4.0) |
| Systolic blood pressure (mmHg), mean (SD) | 134.32 (15.86) | 135.6 (15.3) | 129.5 (17.3) |
| Diastolic blood pressure (mmHg), mean (SD) | 83.75 (9.16) | 84.2 (9.2) | 82.1 (9.0) |
| **Physical activity and sedentary behaviour** |  |  |  |
| Steps/day, mean (SD) | 6570 (3090) | 6183 (2857) | 7937 (3511) |
| MVPA (min/day), mean (SD)^5^ | 29.3 (23.7) | 26.2 (20.9) | 38.4 (28.0) |
| LPA (min/day), mean (SD)^5^ | 220.07 (65.40) | 215.0 (65.8) | 237.7 (61.5) |
| SB (min/day), mean (SD)^5^ | 588.50 (84.88) | 596.9 (81.2) | 563.5 (93.1) |
| Reach PA recommendation of >150 min MVPA/week, n (%) | 94 (53.7) | 69 (50.7) | 26 (65.0) |
