# Supplementary material for: Effects of a three-armed randomised controlled trial using self-monitoring of daily steps with and without counselling in prediabetes and type 2 diabetes—the Sophia Step Study
Source: Int J Behav Nutr Phys Act. 2021 Sep 8;18:121. doi: 10.1186/s12966-021-01193-w (PMC8424865; doi:10.1186/s12966-021-01193-w)
Supplement: Supplementary file 3 — Additional file 3: Table. Description of changes in medications. [file 12966_2021_1193_MOESM3_ESM.docx]

**Description of medications at baseline and changes in medications across the study period.**

| **Multicomponent group** | **Single component group** | **Control group** |
| --- | --- | --- |
| **Baseline**  7 on Insulin treatment (>6 months)  12 on SU  36 on Metformin, Januvia or Victosa  6 on Inkretins  13 on Betablockers  30 on ACE inhibitors or ARB  30 on Statins | **Baseline**  5 on Insulin treatment (>6 months)  5 on SU  31 on Metformin, Januvia or Victosa  5 on Inkretins  15 on Betablockers  24 on ACE inhibitors or ARB  30 on Statins | **Baseline**  11 on Insulin treatment (>6 months)  13 on SU  22 on Metformin, Januvia or Victosa  4 on Inkretins  1 on GLP1analog  2 on SGLT2  18 on Betablockers  35 on ACE inhibitors or ARB  27 on Statins |
| **Baseline – 3 months (including week 8)**  1 Started Actos  1 Started Simvastatin  1 Started Viktoza  1 Stopped Metformin and started Janvmet  1 Stopped Forxiga and started Victoza  1 Increased dose Enalapril  1 Increased dose Metroprolol  1 Increased dose Simvastatin  1 Increased dose Felopidin  2 Reduced dose Metformin  1 Reduced dose Minidiab  1 Reduced dose Losartad, started and reduced Atorvastatin  1 Reduced dose Insulatard | **Baseline – 3 months (including week 8)**  1 Started Feladipin and Metformin  1 Started Spironolakton  1 Started Atorvastatin  1 Started Humalog mix  1 Started Alfuzossin  1 Started Cortisone treatment  1 Stopped Felodipin and started Enalapril  1 Stopped Trombyl  1 Paused Metformin  1 Increased dose Metformin  1 Increased dose Insuman  1 Increased dose Levaxin  1 Increased dose Laritus and Victoza  1 Increased dose Felodipin  1 Reduced dose Prednisolon  1 Reduced dose Digoxin and Levaxin | **Baseline – 3 months (including week 8)**  1 Started Pravastatin  1 Started Amlopidin and Clopidogrel, increased Simvastatin and reduced Ramipril  1 Started Amlodipin  1 Started Simvastatin  2 Stopped Simvastatin  1 Stopped Metoprolol and started Logimax  1 Stopped Simvastatin, started Atorvastatin and reduced dose Metroprol  1 Increased dose insulin  1 Increased dose Amlodipin  1 Reduced dose Enalapril  1 Reduced dose Metformin |
| **3 months – 6 months (including week 16)**  1 Started Predisolon 1 Started with insulin (Hermelin NPA)  1 Stopped Emconcor  1 Increased dose Abasaglar  1 Reduced dose Glibenklamid  1 is under insulin adjustment and started Humalogmix | **3 months – 6 months (including week 16)**  1 Started Enapril  1 Started Amlodipin  1 Started Cortisone treatment  1 Started Amlodipin  1 Stopped Brilique  1 Stopped Victoza  1 Stopped Metformin  1 Increased dose Metformin  1 Reduced dose Metformin  1 Reduced dose Insulin  1 Reduced dose Insvman  1 Reduced dose Atorvastatin  1 Reduced dose Cortisone followed by increased dose Cortisone | **3 months – 6 months (including week 16)**  1 Started Victoza  1 Started Ezetrol  1 Started Imdur, Brilique and Simidon, and increased Enalapril  1 Started Atorvastatin and stopped Simvastatin  1 Stopped Simvastatin  1 Stopped Candesartan  1 reduced dose Losartan |
| **6 months – 12 months (including week 36)**  1 Started Atorvastatadin  1 Started Duroferon and increased Glibenulamid  1 Started Acetylcystin  1 Started Amdipin  1 Started Metformin and reduced Artovastatin  1 Started Metformin  1 Started Clopidogrel and stopped Indur  2 Started Januvia  1 Started Alfadil  1 Stopped Simvastatin  1 Stopped Ramipril and started Candesartan  1 Stopped Losantan  1 Stopped Simvastatin  1 Increased dose Metformin  2 Reduced dose Humalog mix  1 Reduced dose Metformin  1 Reduced dose Enalapril | **6 months – 12 months (including week 36)**  1 Started Glimepirid  1 Started Prednisolon and stopped metformin  1 Started Simvastatin  1 Started Simvastatin and reduced Novomix  1 Started Pronaxen and Jariunia, increased Arnlodipin  1 Started Blood pressure medication  1 Started Metformin  1 Stopped Victoza  1 Stopped Crestor  1 Stopped Simvastatin and started Atrovastatin  1 Stopped Sertralin and started Mirtazapin  1 Stopped cholesterol lowering medication  1 Increased dose Metformin  1 Increased dose Venlafaxine, samma person  2 Reduced dose Metformin  1 Reduced dose Enalapril | **6 months – 12 months (including week 36)**  1 Started Candersartan  1 Started Ezetrol and stopped statins  1 Started Furix  1 Started Victoza, Forxiga and Metformin, stopped Janulet  1 Started Atrovastatin and increased Enalapris  1 Started Clopidogrel  1 Started Hydroklortiazid  1 Started Inolaxol and Cilaxoral  1 Started Braltus  1 Stopped Simvastatin  1 Stopped Cortisone  1 Stopped Metformin and reduced Humalogmix  1 Increased dose Mindiab  1 Increased dose Metformin  1 Reduced dose Enalapril  1 Reduced dose Cortisone  1 Reduced dose Atorvastatin |
| **12 months – 18 months**  1 Started Forxiga  1 Started Metformin and Enalapril  1 Started Victoza  1 Started Humalog mix  1 Started Insuman and stopped Mindiab  1 Started Rosuvastatin  1 Started Metformin  1 Started Losartan  1 Stopped Metformin  1 Stopped Glibeklamid  2 Stopped Amlodipin  1 Increased dose Metformin  1 Reduced dose Metformin and started | **12 months – 18 months**  1 Started Simrastratik  1 Started Jardiance, Victoza, Lantus and Metformin  1 Stopped Metformin  1 Increased dose Metformin  1 Increased dose Losartan  1 Reduced dose Metformin | **12 months – 18 months**  1 Started Victoza  1 Started Civestor, Folacih and Behepen and stopped Simuaststin  1 Started Metformin  1 Started Amlodipin  1 Started Novomix and Ozempic, stopped Trulicity and increased Enalapril  1 Stopped Crestor and Metformin, increased Glimepirid  1 Stopped Humulin and started Humalog  1 Increased dose Furix  1 Increased dose Blood pressure medication |
| **18 months – 24 months**  1 Started Metoformin  1 Started Eliquis  1 Stopped Glimepirid  1 Stopped Besacip and started Atorvastatin  1 Stopped Metformin  1 Stopped Statine  1 Increased dose Atorvastatin  1 Increased dose Krestor and Metoformin  1 Increased dose Metoformin  1 Increased dose Simvastatin  1 Reduced dose Metoformin  1 is under insulin adjustments, diagnosed with LADA | **18 months – 24 months**  1 Started Alfuzosin and increased Metformin  1 Stopped Metformin  1 Stopped Enalapril and started Losartan/Hydrochlorothiazide  1 Stopped Waran and started Eliquis  1 Increased dose Amlodipin  1 Increased dose Insuman  1 Reduced dose Metformin  1 Reduced dose Novomix | **18 months – 24 months**  1 Started Glimepirid and Forxiga, and stopped Metformin  1 Started Metformin  1 Stopped Victoza  1 Stopped Predkisokon  1 Stopped Furix  1 Stopped Simvastatin  1 Increased dose Folacin  2 Increased dose Metformin  1 Increased dose Hydroklortiazid |
