# Supplementary figures and images for: Effects of a three-armed randomised controlled trial using self-monitoring of daily steps with and without counselling in prediabetes and type 2 diabetes—the Sophia Step Study
Source: Int J Behav Nutr Phys Act. 2021 Sep 8;18:121. doi: 10.1186/s12966-021-01193-w (PMC8424865; doi:10.1186/s12966-021-01193-w)

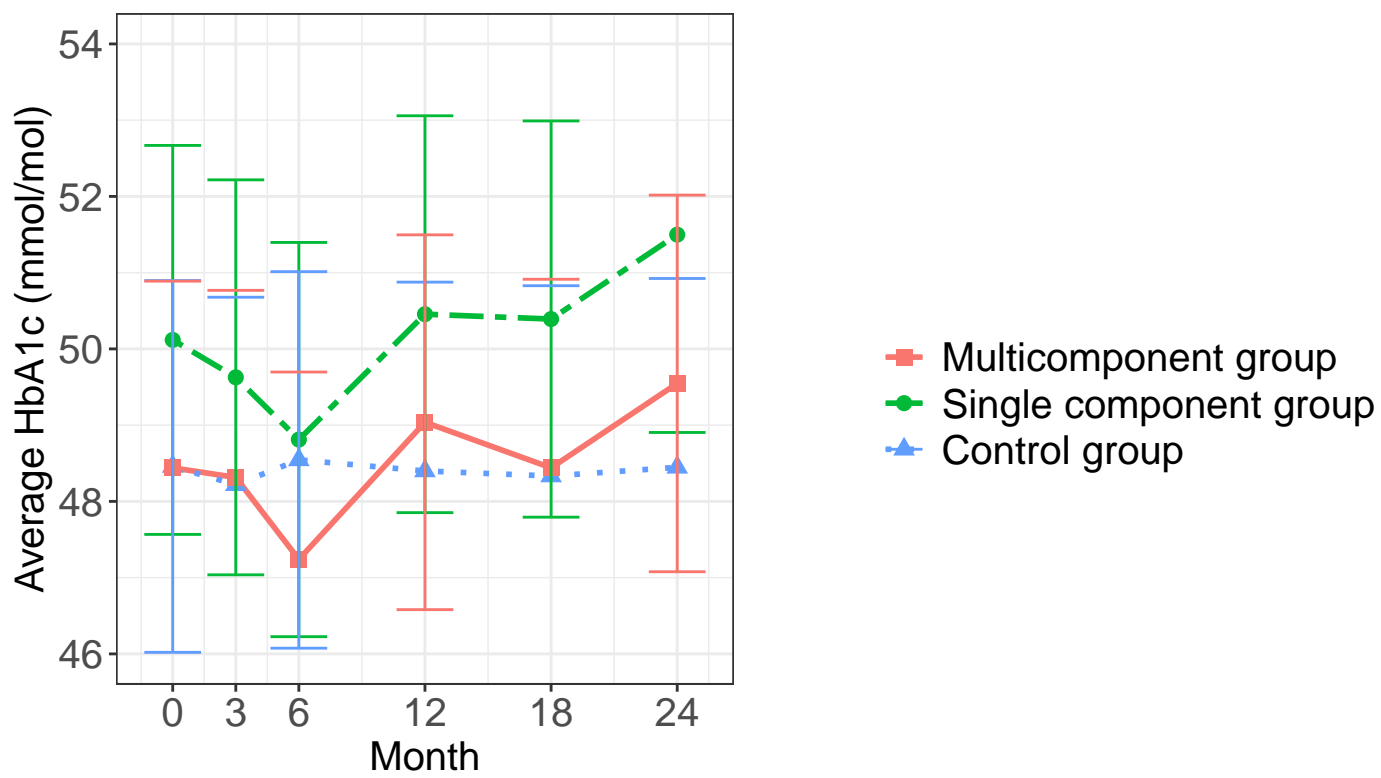

Supplement: Supplementary file 4 — Additional file 4: Figure. Mean change in HbA1c for each group over 24 months with confidence intervals included. [file 12966_2021_1193_MOESM4_ESM.pdf]

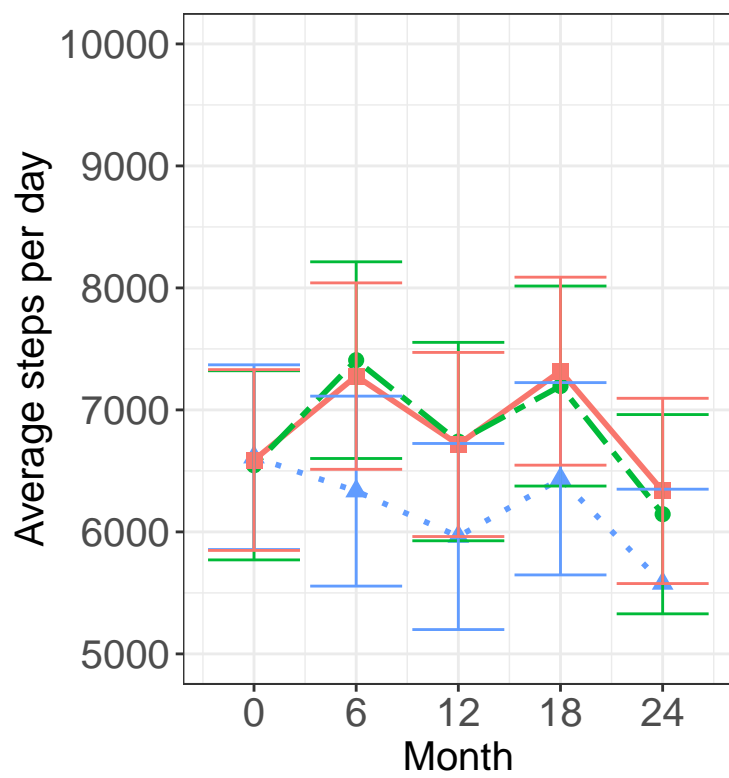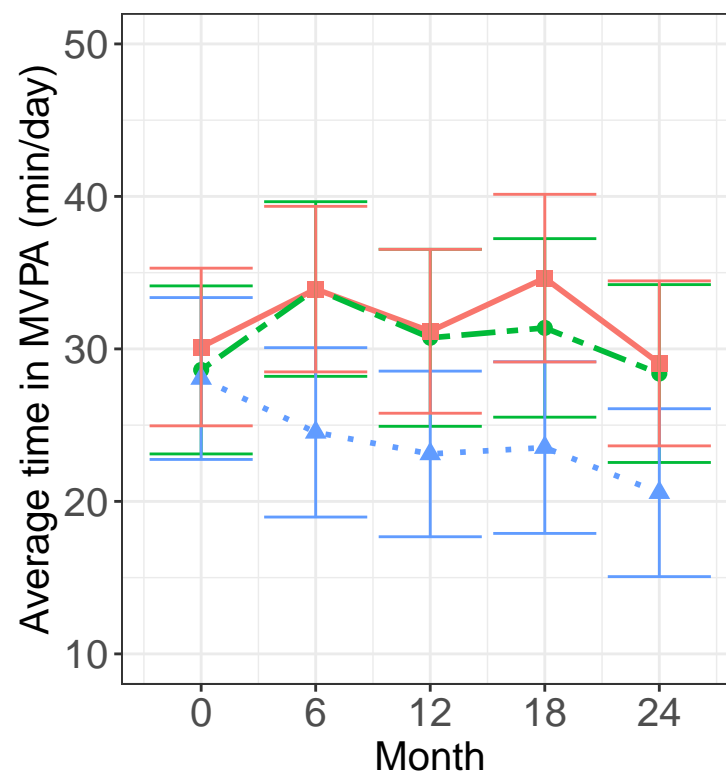

- Multicomponent group
- Single component group
- ▲ Control group

Supplement: Supplementary file 5 — Additional file 5: Figure. Mean change in MVPA and daily steps for each group over 24 months with confidence intervals included. [file 12966_2021_1193_MOESM5_ESM.pdf]
