# Supplementary material for: Effects of a three-armed randomised controlled trial using self-monitoring of daily steps with and without counselling in prediabetes and type 2 diabetes—the Sophia Step Study
Source: Int J Behav Nutr Phys Act. 2021 Sep 8;18:121. doi: 10.1186/s12966-021-01193-w (PMC8424865; doi:10.1186/s12966-021-01193-w)
Supplement: Supplementary file 6 — Additional file 6: Table. Description of changes in each group. [file 12966_2021_1193_MOESM6_ESM.docx]

**Table S1a.** Mean differences of cardiometabolic risk factors between baseline and 3, 6, 12, 18 and 24 months per intervention group.

|  | **Baseline to**  **3 months**  **(95% CI)** | **Baseline to**  **6 months**  **(95% CI)** | **Baseline to**  **12 months**  **(95% CI)** | **Baseline to**  **18 months**  **(95% CI)** | **Baseline to**  **24 months**  **(95% CI)** |
| --- | --- | --- | --- | --- | --- |
| **Multicomponent intervention** |  |  |  |  |  |
| HbA1c (mmol/mol) | -1.1 (-3.2 to 0.9) (n=60) | -2.3 (-4.5 to -0.1) (n=59) | 0.3 (-0.9 to 1.6) (n=59) | -0.2 (-1.8 to 1.3) (n=54) | 1.0 (-0.9 to 2.8) (n=55) |
| Fasting glucose (mmol/L) | 0.3 (0.0 to 0.6) (n=59) | -0.1 (-0.5 to 0.2) (n=59) | 0.0 (-0.5 to 0.5) (n=45) | 0.2 (-0.3 to 0.7) (n=53) | 0.4 (0.0 to 0.7) (n=54) |
| C-Peptide (nmol/L) | 0.06 (-0.02 to 0.14)  (n=56) | -0.04 (-0.12 to 0.04)  (n=62) | 0.02 (-0.06 to 0.09)  (n=60) |  | 0.04 (-0.06 to 0.14)  (n=56) |
| ApoB/ApoA1 | -0.1 (-0.1 to -0.0) (n=10) | -0.1 (-0.1 to 0.0) (n=11) | -0.2 (-0.2 to -0.1) (n=10) |  | -0.1 (-0.2 to 0.0) (n=12) |
| HDL cholesterol (mmol/L) | 0.1 (0.0 to 0.1) (n=60) | 0.0 (0.0 to 0.1) (n=59) | 0.0 (0.0 to 0.1) (n=59) |  | 0.1 (0.00 to 0.1) (n=55) |
| LDL cholesterol (mmol/L) | 0.1 (-0.1 to 0.3) (n=46) | -0.1 (-0.3 to 0.1) (n=46) | -0.3 (-0.5 to -0.0) (n=58) |  | -0.1 (-0.4 to 0.1) (n=54) |
| Triglycerides (mmol/L) | -0.01 (-0.19 to 0.16)  (n=60) | -0.19 (-0.32 to -0.06)  (n=60) | -0.16 (-0.29 to -0.03)  (n=59) |  | -0.14 (-0.31 to 0.02)  (n=55) |
| Total cholesterol (mmol/L) | 0.12 (-0.10 to 0.35)  (n=48) | -0.13 (-0.33 to 0.07)  (n=47) | -0.31 (-0.56 to -0.07)  (n=59) |  | -0.18 (-0.48 to 0.11)  (n=55) |
| Systolic blood pressure (mmHg) | -2.9 (-6.5 to 0.7) (n=64) | -1.9 (-5.7 to 2.0) (n=63) | -2.1 (-5.4 to 1.2) (n=60) | -1.3 (-4.9 to 2.2) (n=58) | 1.9 (-1.8 to 5.7) (n=57) |
| Diastolic blood pressure (mmHg) | -2.6 (-4.6 to -0.5) (n=64) | -2.1 (-4.4 to 0.1) (n=63) | -2.4 (-4.7 to -0.1) (n=60) | -2.1 (-4.7 to 0.4) (n=58) | 2.2 (-0.7 to 5.1) (n=58) |
|  |  |  |  |  |  |
| **Single component intervention** |  |  |  |  |  |
| HbA1c (mmol/mol) | -1.4 (-3.4 to 0.5) (n=50) | -2.3 (-4.5 to -0.1) (n=51) | 0.5 (-1.9 to 2.8) (n=47) | -0.1 (-2.4 to 2.2) (n=48) | 1.6 (-1.2 to 4.3) (n=49) |
| Fasting glucose (mmol/L) | -0.3 (-0.8 to 0.2) (n=50) | -0.1 (-0.5 to 0.4) (n=51) | 0.1 (-0.2 to 0.4) (n=34) | -0.0 (-0.7 to 0.6) (n=49) | -0.2 (-0.7 to 0.4) (n=49) |
| C-Peptide (nmol/L) | -0.07 (-0.15 to 0.02)  (n=51) | -0.01 (-0.08 to 0.05)  (n=52) | -0.02 (-0.10 to 0.07)  (n=51) |  | 0.01 (-0.06 to 0.09)  (n=48) |
| ApoB/ApoA1 | -0.1 (-0.1 to 0.0) (n=14) | 0.0 (-0.1 to 0.0) (n=16) | 0.0 (-0.1 to 0.1) (n=14) |  | 0.0 (-0.1 to 0.1) (n=16) |
| HDL cholesterol (mmol/L) | 0.0 (-0.1 to 0.0) (n=50) | 0.0 (0.0 to 0.1) (n=51) | 0.00 (-0.1 to 0.1) (n=46) |  | 0.0 (0.0 to 0.1) (n=49) |
| LDL cholesterol (mmol/L) | 0.0 (-0.2 to 0.2) (n=34) | 0.0 (-0.3 to 0.2) (n=32) | 0.1 (0.1 to 0.3) (n=45) |  | 0.0 (-0.2 to 0.2) (n=47) |
| Triglycerides (mmol/L) | -0.14 (-0.30 to 0.02)  (n=49) | -0.31 (-0.54 to -0.08)  (n=51) | -0.22 (-0.47 to 0.03)  (n=47) |  | -0.07 (-0.35 to 0.21)  (n=49) |
| Total cholesterol (mmol/L) | 0.02 (-0.19 to 0.22)  (n=35) | -0.11 (-0.39 to 0.17)  (n=34) | -0.03 (-0.26 to 0.20)  (n=47) |  | 0.08 (-0.18 to 0.33)  (n=49) |
| Systolic blood pressure (mmHg) | -5.1 (-8.6 to -1.6) (n=52) | -4.8 (-8.7 to -0.8) (n=53) | -4.8 (-8.3 to -1.2) (n=53) | -6.2 (-10.6 to -1.8) (n=48) | -4.1 (-8.6 to 0.4) (n=50) |
| Diastolic blood pressure (mmHg) | -2.3 (-4.4 to -0.1) (n=52) | -2.2 (-4.6 to 0.2) (n=53) | -3.3 (-5.9 to -0.6) (n=53) | -3.6 (-6.2 to -1.0) (n=48) | -3.2 (-6.0 to -0.4) (n=50) |
| **Control group** |  |  |  |  |  |
| HbA1c (mmol/mol) | -1.1 (-2.9 to 0.7) (n=59) | -1.1 (-2.9 to 0.8) (n=56) | -0.9 (-2.9 to 1.2) (n=54) | -1.2 (-3.2 to 0.7)  (n=49) | -0.4 (-2.5 to 1.6)  (n=54) |
| Fasting glucose (mmol/L) | -0.1 (-0.4 to 0.2) (n=58) | 0.1 (-0.3 to 0.5) (n=55) | -0.2 (-0.7 to 0.3) (n=37) | 0.0 (-0.5 to 0.5)  (n=50) | 0.1 (-0.3 to 0.6)  (n=53) |
| C-Peptide (nmol/L) | 0.04 (-0.04 to 0.12)  (n=59) | 0.02 (-0.04 to 0.08)  (n=58) | -0.01 (-0.09 to 0.06)  (n=56) |  | -0.01 (-0.09 to 0.07)  (n=56) |
| ApoB/ApoA1 | 0.0 (-0.1 to 0.00) (n=17) | -0.1 (-0.2 to 0.0) (n=18) | -0.1 (-0.1 to 0.0) (n=17) |  | 0.0 (-0.1 to 0.1) (n=16) |
| HDL cholesterol (mmol/L) | 0.0 (-0.1 to 0.0) (n=58) | 0.0 (-0.1 to 0.1) (n=57) | 0.1 (0.00 to 0.2) (n=54) |  | 0.1 (0.0 to 0.1) (n=55) |
| LDL cholesterol (mmol/L) | -0.2 (-0.5 to 0.1) (n=41) | -0.2 (-0.5 to 0.1) (n=38) | **-**0.2 (-0.5 to 0.00) (n=52) |  | -0.3 (-0.5 to 0.0) (n=51) |
| Triglycerides (mmol/L) | 0.12 (-0.09 to 0.33)  (n=58) | -0.01 (-0.29 to 0.27)  (n=57) | -0.04 (-0.26 to 0.19)  (n=54) |  | -0.17 (-0.46 to 0.11)  (n=54) |
| Total cholesterol (mmol/L) | -0.20 (-0.56 to 0.17)  (n=42) | -0.22 (-0.53 to 0.08)  (n=40) | 0.68 (-1.16 to 2.51)  (n=54) |  | -0.22 (-0.52 to 0.09)  (n=54) |
| Systolic blood pressure (mmHg) | -2.8 (-6.9 to 1.2) (n=61) | -2.9 (-6.8 to 1.1) (n=59) | -1.5 (-6.2 to 3.1) (n=57) | -3.1 (-8.0 to 1.8) (n=54) | -2.8 (-7.0 to 1.3) (n=54) |
| Diastolic blood pressure (mmHg) | 0.3 (-2.0 to 2.6) (n=61) | -0.3 (-2.9 to 2.3) (n=59) | -0.8 (-3.5 to 2.0) (n=57) | -2.7 (-5.6 to 0.2) (n=54) | -2.3 (-5.2 to 0.6) (n=54) |

**Table S1b.** Mean differences of anthropometry between baseline and 3, 6, 12, 18 and 24 months per intervention group.

|  | **Baseline to**  **3 months**  **(95% CI)** | **Baseline to**  **6 months**  **(95% CI)** | **Baseline to**  **12 months**  **(95% CI)** | **Baseline to**  **18 months**  **(95% CI)** | **Baseline to**  **24 months**  **(95% CI)** |
| --- | --- | --- | --- | --- | --- |
| **Multicomponent intervention** |  |  |  |  |  |
| Sagittal abdominal diameter (cm) | -0.2 (-0.6 to 0.2) (n=63) | -0.4 (-0.8 to 0.0) (n=62) | -0.5 (-0.9 to -0.1) (n=59) |  | -0.1 (-0.6 to 0.3) (n=56) |
| Weight (kg) | -0.2 (-0.7 to 0.4) (n=64) | -1.0 (-1.7 to -0.3) (n=63) | -0.5 (-1.4 to 0.5) (n=61) | -0.8 (-1.9 to 0.2) (n=58) | -0.1 (-1.0 to 0.8) (n=57) |
| Body Mass Index (kg/m^2^) | 0.0 (-0.2 to 0.3) (n=64) | -0.3 (-0.6 to 0.00) (n=63) | -0.1 (-0.6 to 0.3) (n=61) | -0.3 (-0.8 to 0.1) (n=58) | 0.0 (-0.4 to 0.4) (n=57) |
| Body fat (%) | -0.5 (-1.0 to -0.1) (n=64) | -1.1 (-1.6 to -0.6) (n=62) | -0.5 (-1.1 to 0.1) (n=61) | -1.0 (-1.8 to -0.3) (n=58) | -0.1 (-0.7 to 0.5) (n=56) |
| Waist circumference (cm) | -0.8 (-1.5 to 0.0)  (n=64) | -1.9 (-2.8 to -1.1)  (n=63) | -1.8 (-2.8 to -0.8)  (n=60) | -1.41 (-2.5 to -0.3)  (n=58) | -1.0 (-2.2 to 0.2)  (n=57) |
| **Single component intervention** |  |  |  |  |  |
| Sagittal abdominal diameter (cm) | -0.3 (-0.6 to 0.1) (n=52) | -0.3 (-0.8 to 0.2) (n=53) | -0.5 (-1.1 to 0.0) (n=52) |  | -0.2 (-0.9 to 0.5) (n=50) |
| Weight (kg) | -0.8 (-1.4 to -0.1) (n=52) | -1.3 (-2.2 to -0.4) (n=53) | -1.3 (-2.3 to -0.3) (n=53) | -1.4 (-2.6 to -0.3) (n=49) | -1.0 (-2.2 to 0.3) (n=51) |
| Body Mass Index (kg/m^2^) | -0.1 (-0.5 to 0.2) (n=52) | -0.4 (-0.8 to 0.0) (n=53) | -0.3 (-0.8 to 0.1) (n=53) | -0.4 (-0.9 to 0.1) (n=49) | -0.2 (-0.6 to 0.3) (n=51) |
| Body fat (%) | 0.0 (-0.6 to 0.6) (n=51) | -0.3 (-1.1 to 0.4) (n=53) | -0.4 (-1.1 to 0.4) (n=53) | -0.6 (-1.5 to 0.3) (n=49) | -0.4 (-1.3 to 0.5) (n=51) |
| Waist circumference (cm) | -0.7 (-1.6 to 0.2)  (n=51) | -1.4 (-2.4 to -0.5)  (n=53) | -1.5 (-2.7 to -0.3)  (n=53) | -1.5 (-3.1 to 0.0)  (n=49) | -0.8 (-2.4 to 0.9)  (n=51) |
| **Control group** |  |  |  |  |  |
| Sagittal abdominal diameter (cm) | -0.4 (-0.7 to -0.1) (n=60) | -0.4 (-0.7 to 0.0) (n=58) | -0.6 (-1.1 to -0.2) (n=56) |  | -0.2 (-0.7 to 0.3) (n=51) |
| Weight (kg) | -0.6 (-1.2 to 0.0) (n=61) | -0.8 (-1.6 to 0.0) (n=59) | -1.0 (-2.3 to 0.4) (n=57) | -1.5 (-3.4 to 0.4) (n=54) | -1.5 (-3.3 to 0.4) (n=55) |
| Body Mass Index (kg/m^2^) | -0.1 (-0.4 to 0.1) (n=61) | -0.2 (-0.5 to 0.1) (n=59) | -0.3 (-0.7 to 0.2) (n=57) | -0.6 (-1.2 to 0.0) (n=54) | -0.5 (-1.1 to 0.2) (n=55) |
| Body fat (%) | -0.2 (-0.7 to 0.3) (n=60) | -0.2 (-0.7 to 0.3) (n=58) | -0.3 (-1.0 to 0.4) (n=57) | -0.8 (-1.5 to -0.1) (n=53) | -0.2 (-1.2 to 0.7) (n=55) |
| Waist circumference (cm) | -1.3 (-2.2 to -0.4)  (n=61) | -0.2 (-3.8 to 3.3)  (n=59) | -2.7 (-3.9 to -1.5)  (n=57) | -2.8 (-4.2 to -1.3)  (n=54) | -2.0 (-3.4 to -0.5)  (n=55) |

**Table S1c.** Mean differences of Physical activity and sedentary behaviour between baseline and 6, 12, 18 and 24 months, per intervention group.

|  | **Baseline to**  **6 months**  **(95% CI)** | **Baseline to**  **12 months**  **(95% CI)** | **Baseline to**  **18 months**  **(95% CI)** | **Baseline to**  **24 months**  **(95% CI)** |
| --- | --- | --- | --- | --- |
| **Multicomponent intervention** |  |  |  |  |
| MVPA | 5.5 (0.3 to 10.7) (n=48) | 0.2 (-4.3 to 4.6) (n=50) | 4.1 (-0.7 to 8.8) (n=44) | -1.5 (-6.2 to 3.3) (n=48) |
| LPA | 10.8 (-1.7 to 23.3) (n=48) | 4.3 (-6.2 to 14.8) (n=50) | 20.0 (3.6 to 36.5) (n=44) | 1.5 (-14.8 to 17.8) (n=48) |
| SB | -2.6 (-26.7 to 21.5) (n=48) | -16.2 (-35.1 to 2.8) (n=50) | -21.4 (-44.9 to 2.1) (n=44) | 1.0 (-30.2 to 32.2) (n=48) |
| Daily steps | 876 (155 to 1597)  (n=40) | 89 (-446 to 625)  (n=45) | 959 (306 to 1610)  (n=39) | -208 (-875 to 459)  (n=42) |
| **Single component intervention** |  |  |  |  |
| MVPA | 5.5 (-1.7 to 12.6) (n=44) | 2.8 (-2.5 to 8.0) (n=41) | 4.0 (-4.2 to 12.3) (n=38) | 0.8 (-5.2 to 6.8) (n=40) |
| LPA | 17.5 (3.8 to 31.1) (n=44) | 3.0 (-11.2 to 17.3) (n=41) | 18.7 (0.2 to 37.2) (n=38) | -15.6 (-30.0 to -1.2)  (n=40) |
| SB | -11.6 (-26.4 to 3.3) (n=44) | -6.4 (-27.2 to 14.4) (n=41) | -13.4 (-38.9 to 12.2) (n=38) | 12.7 (-7.1 to 32.4) (n=40) |
| Daily steps | 1009 (166 to 1852) (n=43) | 272 (-396 to 939) (n=41) | 830 (26 to 1634) (n=38) | -295 (-950 to 360) (n=40) |
| **Control group** |  |  |  |  |
| MVPA | -6.4 (-11.0 to -1.8) (n=41) | -7.1 (-11.3 to -2.8) (n=46) | -8.6 (-16.2 to -1.0) (n=39) | -9.9 (-14.8 to -4.9) (n=43) |
| LPA | 11.3 (-2.6 to 25.1) (n=41) | -5.5 (-19.4 to 8.3) (n=46) | 14.4 (0.1 to 28.8) (n=39) | -5.9 (-20.3 to 8.6) (n=43) |
| SB | 5.1 (-20.2 to 30.5) (n=41) | 37.5 (0.7 to 74.3) (n=46) | 18.4 (-14.6 to 51.5) (n=39) | 14.1 (-12.8 to 41.1) (n=43) |
| Daily steps | -503 (-1116 to 110)  (n=37) | -948 (-1535 to -361)  (n=41) | -440 (-1372 to 492)  (n=35) | -1295 (-1940 to -650)  (n=38) |

MVPA = Time in Moderate to Vigorous Physical Activity, LPA = Time in Light intensity Physical Activity, SB = Time in Sedentary Behavior.
