# Supplementary material for: Effects of a three-armed randomised controlled trial using self-monitoring of daily steps with and without counselling in prediabetes and type 2 diabetes—the Sophia Step Study
Source: Int J Behav Nutr Phys Act. 2021 Sep 8;18:121. doi: 10.1186/s12966-021-01193-w (PMC8424865; doi:10.1186/s12966-021-01193-w)
Supplement: Supplementary file 7 — Additional file 7: Table. Effects on secondary outcomes. [file 12966_2021_1193_MOESM7_ESM.docx]

**Table S1. Intervention effect over time between interventions and control groups.**

|  | **Intervention effect at**  **3 months**  **(95% CI)** | **Intervention effect at**  **6 months**  **(95% CI)** | **Intervention effect at 12 months**  **(95% CI)** | **Intervention effect at 18 months**  **(95% CI)** | **Intervention effect at**  **24 months**  **(95% CI)** |
| --- | --- | --- | --- | --- | --- |
| **Multicomponent intervention vs control group** |  |  |  |  |  |
| Fasting glucose (mmol/L) | 0.2 (-0.3 to 0.8) | -0.1 (-0.7 to 0.5) | 0.1 (-0.5 to 0.7) | 0 (-0.6 to 0.6) | 0.2 (-0.4 to 0.8) |
| C-Peptide (nmol/L) | 0.07 (-0.07 to 0.21) | -0.06 (-0.20 to 0.08) | 0.01 (-0.13 to 0.15) |  | 0.01 (-0.13 to 0.15) |
| ApoB/ApoA1 | -0.0 (-0.1 to 0.1) | 0.0 (-0.1 to 0.1) | -0.1 (-0.2 to 0.1) |  | 0.0 (-0.1 to 0.1) |
| HDL cholesterol (mmol/L) | 0.0 (-0.1 to 0.2) | 0 (-0.2 to 0.1) | -0.1 (-0.2 to 0.1) |  | -0.0 (-0.2 to 0.1) |
| LDL cholesterol (mmol/L) | 0.1 (-0.2 to 0.5) | 0.0 (-0.3 to 0.4) | -0.1 (-0.4 to 0.3) |  | 0.1 (-0.2 to 0.5) |
| Triglycerides (mmol/L) | 0.06 (-0.19 to 0.3) | -0.07 (-0.31 to 0.17) | 0.08 (-0.16 to 0.32) |  | 0.15 (-0.09 to 0.40) |
| Total cholesterol (mmol/L) | 0.21 (-0.18 to 0.6) | -0.01 (-0.40 to 0.38) | -0.16 (-0.53 to 0.22) |  | 0.08 (-0.30 to 0.45) |
| Systolic blood pressure (mmHg) | -1.8 (-6.6 to 3.0) | -2.5 (-7.3 to 2.3) | -2.8 (-7.7 to 2.0) | -0.2 (-5.2 to 4.7) | 0.7 (-4.3 to 5.7) |
| Diastolic blood pressure (mmHg) | -2.9 (-6.0 to 0.1) | -2.4 (-5.4 to 0.7) | -1.8 (-4.9 to 1.3) | 0.2 (-2.9 to 3.4) | 2.9 (-0.21to 6.1) |
| **Single component intervention vs control group** |  |  |  |  |  |
| Fasting glucose (mmol/L) | 0.1 (-0.5 to 0.7) | 0.3 (-0.4 to 0.9) | 0.2 (-0.4 to 0.9) | 0.1 (-0.5 to 0.7) | 0.1 (-0.5 to 0.7) |
| C-Peptide (nmol/L) | -0.17 (-0.32 to -0.03) | -0.15 (-0.30 to -0.01) | -0.13 (-0.27 to 0.02) |  | -0.11 (-0.25 to 0.04) |
| ApoB/ApoA1 | 0.0 (-0.1 to 0.1) | 0.0 (-0.1 to 0.1) | 0.0 (-0.1 to 0.1) |  | 0 (-0.1 to 0.1) |
| HDL cholesterol (mmol/L) | -0.1 (-0.3 to 0.1) | -0.1 (-0.2 to 0.1) | -0.1 (-0.3 to 0.0) |  | -0.1 (-0.3 to 0.0) |
| LDL cholesterol (mmol/L) | 0.1 (-0.3 to 0.5) | 0.0 (-0.4 to 0.3) | 0.2 (-0.2 to 0.5) |  | 0.2 (-0.2 to 0.5) |
| Triglycerides (mmol/L) | -0.04 (-0.29 to 0.21) | -0.12 (-0.37 to 0.13) | 0.05 (-0.20 to 0.31) |  | 0.17 (-0.09 to 0.42) |
| Total cholesterol (mmol/L) | 0.02 (-0.39 to 0.43) | -0.20 (-0.61 to 0.21) | -0.07 (-0.46 to 0.32) |  | 0.03 (-0.36 to 0.41) |
| Systolic blood pressure (mmHg) | 2.3 (-2.8 to 7.3) | 2.1 (-2.9 to 7.1) | 1.1 (-3.9 to 6.2) | 1.3 (-3.8 to 6.5) | 2.7 (-2.4 to 7.9) |
| Diastolic blood pressure (mmHg) | -0.2 (-3.4 to 3.0) | 0.1 (-3.1 to 3.2) | -0.2 (-3.4 to 3.0) | 0.9 (-2.4 to 4.1) | 1.2 (-2.0 to 4.5) |

Effect sizes and 95% confidence interval (CI) for cardiometabolic risk factors from the robust repeated linear mixed model analysis.

**Table S2.** **Intervention effect over time between interventions and control groups.**

|  | | **Intervention effect at**  **3 months**  **(95% CI)** | | **Intervention effect at**  **6 months**  **(95% CI)** | | **Intervention effect at 12 months**  **(95% CI)** | | **Intervention effect at 18 months**  **(95% CI)** | | **Intervention effect at**  **24 months**  **(95% CI)** | |
| --- | --- | --- | --- | --- | --- | --- | --- | --- | --- | --- | --- |
| **Multicomponent intervention vs control group** | |  | |  | |  | |  | |  | |
| Sagittal abdominal diameter (cm) | | 0.3 (-1.0 to 1.7) | | 0.2 (-1.2 to 1.5) | | 0.2 (-1.2 to 1.5) | |  | | 0.3 (-1.0 to 1.7) | |
| Weight (kg) | | -1.0 (-6.6 to 4.5) | | -1.7 (-7.2 to 3.9) | | -1.2 (-6.7 to 4.3) | | -1.6 (-7.1 to 4.0) | | -0.5 (-6.1 to 5.0) | |
| Body Mass Index (kg/m^2^) | | 0.6 (-0.9 to 2.1) | | 0.4 (-1.1 to 1.9) | | 0.4 (-1.1 to 2.0) | | 0.4 (-1.1 to 1.9) | | 0.7 (-0.8 to 2.2) | |
| Body fat (%) | | 1.7 (-1.4 to 4.7) | | 1.4 (-1.7 to 4.4) | | 1.7 (-1.3 to 4.7) | | 1.8 (-1.3 to 4.8) | | 2.2 (-0.9 to 5.2) | |
| Waist circumference (cm) | 1.0  (-3.2 to 5.1) | | 0.5  (-3.7 to 4.7) | | 1.2  (-3.0 to 5.4) | | 1.4  (-2.8 to 5.6) | | 1.0  (-3.2 to 5.2) | |  |
| **Single component intervention vs control group** | |  | |  | |  | |  | |  | |
| Sagittal abdominal diameter (cm) | | -0.2 (-1.6 to 1.2) | | -0.1 (-1.5 to 1.3) | | -0.2 (-1.6 to 1.2) | |  | | -0.2 (-1.7 to 1.2) | |
| Weight (kg) | | -3.8 (-9.5 to 1.8) | | -4.0 (-9.7 to 1.7) | | -4.1 (-9.7 to 1.6) | | -3.8 (-9.5 to 1.8) | | -3.4 (-9.1 to 2.2) | |
| Body Mass Index (kg/m^2^) | | -0.3 (-1.9 to 1.2) | | -0.4 (-2.0 to 1.1) | | -0.4 (-2.0 to 1.1) | | -0.2 (-1.8 to 1.3) | | -0.2 (-1.7 to 1.4) | |
| Body fat (%) | | -0.2 (-3.3 to 2.9) | | -0.3 (-3.4 to 2.8) | | -0.4 (-3.5 to 2.7) | | -0.2 (-3.3 to 2.9) | | -0.4 (-3.5 to 2.7) | |
| Waist circumference (cm) | -1.7  (-5.99 to 2.60) | | -1.4  (-5.67 to 2.91) | | -0.7  (-4.97 to 3.62) | | -0.8  (-5.08 to 3.53) | | -1.0  (-5.30 to 3.30) | |  |

Effect sizes and 95% confidence interval (CI) for anthropometry from the robust repeated linear mixed model analysis.
